# Structures of the DfsB Protein Family Suggest a Cationic, Helical Sibling Lethal Factor Peptide

**DOI:** 10.1016/j.jmb.2016.01.013

**Published:** 2016-02-13

**Authors:** Jonathan D. Taylor, Gabrielle Taylor, Stephen A. Hare, Steve J. Matthews

**Affiliations:** Department of Life Sciences, Imperial College London, London SW7 2AZ, United Kingdom

**Keywords:** Fratricide, sporulation, patterning, bacteriocin, DfsB

## Abstract

Bacteria have developed a variety of mechanisms for surviving harsh environmental conditions, nutrient stress and overpopulation. *Paenibacillus dendritiformis* produces a lethal protein (Slf) that is able to induce cell death in neighbouring colonies and a phenotypic switch in more distant ones. Slf is derived from the secreted precursor protein, DfsB, after proteolytic processing. Here, we present new crystal structures of DfsB homologues from a variety of bacterial species and a surprising version present in the yeast *Saccharomyces cerevisiae*. Adopting a four-helix bundle decorated with a further three short helices within intervening loops, DfsB belongs to a non-enzymatic class of the DinB fold. The structure suggests that the biologically active Slf fragment may possess a C-terminal helix rich in basic and aromatic residues that suggest a functional mechanism akin to that for cationic antimicrobial peptides.

## Highlights

• Crystal structure of DfsB reveals a non-enzymatic variant of the DinB fold.
• Structural features are conserved in prokaryotic and eukaryotic DfsB homologues.
• The sibling lethal factor processing site is highly solvent exposed and within a flexible loop.
• The mature sibling lethal factor resembles a cationic antimicrobial peptide.

## Graphical abstract

**Figure:**
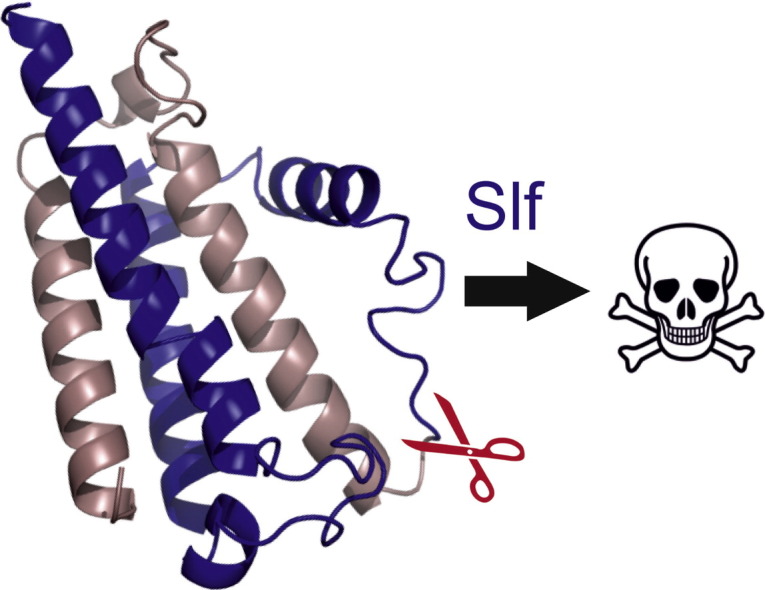

Bacteria possess a variety of mechanisms for survival under harsh environmental conditions, nutrient stress and overpopulation. Furthermore, some bacteria display a remarkable ability to grow into complex patterns [1], [2], [3]. The reason for this and the underlying molecular mechanisms involved remain unknown. *Paenibacillus* are exemplary in this regard, forming a variety of morphotypes on semi-solid agar: chiral branches, swirls and vortices [4]. *Paenibacillus* *dendritiformis* adopts two distinct growth patterns termed Chiral and Branching (tip splitting). Moreover, when the same stock of cells is spotted onto an agar plate a few centimetres apart, the colonies grow outwards evenly in all directions but minimally in the direction of the opposite colony [5]. Clearly, these colonies sense one another and prefer to leave the intervening nutrient-rich region untouched. A 12-kDa polypeptide was shown to be localised exclusively to this region and is derived from a precursor protein termed DfsB (PdDfsB) [5], [6]. This mature polypeptide could be purified from the agar between multiple growing colonies. When applied adjacent to a single colony, the cells nearest died and no growth occurred over several days in rich medium when mixed with planktonic cells. Thus, the fragment was named sibling lethal factor (Slf) as it was associated with cell death and was stimulated by proximity to clonal cells. Later, it was shown that Slf was unable to induce cell death in species outside of *Paenibacillus* [6]. *Bacillus subtilis* cells were immune to its effects, which may suggest a degree of specificity for Slf action.

The precursor PdDfsB is cleaved by subtilisin to yield biologically active Slf, and the chemical gradients of both proteins between two growing colonies could be accurately modelled. In summary, the two advancing colony fronts release subtilisin and DfsB, and as they approach one another, the concentrations of these proteins exceed a threshold and the subsequent formation of Slf induces the death of nearby cells and sporulation in those further away. Given the specificity and pattern-inducing phenotype associated with Slf, one might expect it to be limited to species that display these complex growth behaviours. However, remarkably, the *dfsB* gene is widely distributed across Gram-positive and to some extent Gram-negative bacterial kingdoms (PFAM entry DUF1706). Moreover, the presence of a close homologue of *dfsB* in *Saccharomyces cerevisiae* hints that yeast can influence the viability of co-cultured bacilli.

At present, there are several unanswered questions concerning DfsB/Slf. Foremost is the mechanism by which Slf induces the death of nearby cells. To help understand structural changes upon DfsB/Slf conversion, we crystallised recombinant, His-tagged PdDfsB and solved its atomic structure using X-ray crystallography (Table 1). PdDfsB adopts a four-helical bundle core structure that belongs to the DinB structural superfamily (Fig. 1). This structural clan consists of eight, poorly annotated sub-groups that are found within a very diverse range of bacteria. The family is defined by structure similarity to the *B*. *subtilis* protein DinB (DNA-damage inducible) and the ability to coordinate a metal via conserved histidines (Fig. 1). It is believed that many of the DinB family members are putative metalloenzymes or thiol/glutathione *S*-transferases with many existing structures indicate a dimeric topology [7]. PdDfsB does not follow this pattern: it is a monomer and lacks the conserved residues responsible for coordinating metal ions. Also uniquely, the C-terminal helix of PdDfsB contains a conserved proline that introduces a kink in the helix. Finally, PdDfsB displays a strong positively charge patch that is, again, absent from other DinB family members (Fig. 1). Coupled with the fact that PdDfsB is apparently secreted and processed extracellularly, our structural data suggest that it is not an enzyme.

It was shown that PdDfsB is cleaved by subtilisin, resulting in a 12-kDa fragment termed Slf. The processing site for subtilisin is highly solvent exposed and found within a flexible loop (Fig. 1c). Cleavage by subtilisin results in one of the central α-helices being removed, which would force the remaining polypeptide to collapse to a new state, or oligomerise, or bind other hydrophobic surfaces such as a lipid bilayer. The remaining sequence of Slf is shown in Fig. 1e, and it includes many conserved positive charges and aromatic residues. Although PdDfsB is a well-expressed protein, the production of stable, active samples of the Slf peptide failed despite employing multiple approaches. Firstly, despite extensive effort, we were not able to generate pure Slf via controlled proteolysis of DfsB using commercially available subtilisin in a cleavage reaction. Instead, we observed cleavage at many sites (data not shown), which is likely due to suboptimal *in vitro* reactions conditions. Furthermore, attempts to produce Slf recombinantly through direct expression also failed due to either toxicity during expression or an insoluble product despite the inclusion of a variety of solubility-enhancing tags. Refolding approaches were not successful. Our overall experience suggests that Slf is unstable and highly aggregative when produced in isolation, which likely relates to its prediction function as a membrane binding antimicrobial peptide.

The initial study on Slf reported that it was only active against *Paenibacillus* species; it had no effect on the closely related *B*. *subtilis*. The latter species lacks a DfsB/Slf homologue and it was concluded that the mechanism of the siblicide was host specific. In fact, given its predicted role in reducing intra-species competition by restricting growth, resulting in unique pattern forming on agar plates, one might expect that the *dfsB* gene would be restricted to *Paenibacillu*s. Yet surprisingly, a very large range of bacteria possess a homologue of DfsB/Slf. As noted previously, there is even a homologue (*irc4*) within the yeast genome. We explored the phylogenetic distribution of these DUF1706 family sequences, using CLANS to perform pair-wise alignments and visualise the various clades (Fig. 2). We identify three main clades within DUF1706, two of which are more closely related to each other than to a third clade. The first of these two contain many homologues from Gram-positive bacteria, including pathogenic species, and also the yeast DfsB homologue, IRC4. This protein from *S*. *cerevisiae* is most closely related to the *Lactobacillus* DfsB homologue, implying a long history of association between these disparate organisms. An intriguing question is whether yeast secretes a processed form of IRC4, analogous to Slf, which goes on to control the population of co-cultured *Lactobacillus* species. The second, related clade contains DfsB homologues from mainly Gram-negative bacteria, again including many pathogens. The third, well-populated clade contains exclusively Gram-positive bacteria such as *Listeria* and some *Streptococcus* species. There are also minor sequence clusters from α-proteobacteria and Actinobacteria; however, these are represented by only a few sequences. We also constructed a phylogenetic tree using 57 representative sequences [8], [9], which recapitulates the genetic relationships within the DUF1706 family described above (Fig. S1).

We sought to characterise a selected subset of DfsB homologues structurally, focussing on the three main clades shown in Fig. 2. We were able to express, purify and obtain crystal structures for DfsB homologues from *Clostridium difficile* 630 and *Escherichia coli* UTI89, as well as of IRC4 from *S*. *cerevisiae* (Fig. 3). For the purposes of clarity, we refer to the first two proteins as CdDfsB and EcDfsB, respectively; however, we have no evidence that they are truly acting as DfsB homologues by undergoing cleavage and release of their analogous Slf “toxin”. We observed no overexpression of DfsB homologues from the third main clade (e.g., *Streptococcus sanguinis* or *Streptococcus gordonii*). The overall structures of CdDfsB, EcDfsB and IRC4 are identical with that of PdDfsB, with strong conservation of overall topology and surface charge, particularly in the C-terminal helix (Fig. S2).

During purification of the two *Streptococcus* DfsB homologues (*Streptococcus agalactiae* and *Streptococcus pneumoniae*), both monomers and stable dimers could be identified (Fig. 4). We attempted to crystallise both forms independently and only the dimers yielded diffraction-quality crystals. We solved the crystal structure of a *S*. *agalactiae* DfsB dimer (SaDfsB; Fig. 4) by molecular replacement using a pruned CdDfsB structure as the search model. Interestingly, the source of dimerisation is via the C-terminal helix, which flips out of one monomer and inserts into the other in a mutual fashion.

We found that some species, for example, *Streptococcus mutans*, do not possess the full DfsB precursor but, instead, express a shorter polypeptide that is essentially the isolated Slf fragment. Although it remains possible that these are pseudogenes, it is intriguing to consider the fact that DfsB/Slf is highly specific towards its own species and that many bacteria—including those that are human pathogens—possess a DfsB homologue. The most strongly conserved region of PdDfsB is the C-terminal helix, which is present within the mature Slf sequence. The pattern of conserved positive charge and aromatic positions (Fig. 1f) is reminiscent of cationic antimicrobial peptides, which damage cell membranes [10], [11], [12]. In this scenario, if the Slf fragment retained its amphipathic helical conformation, it would present basic and hydrophobic residues for interaction with the bacterial membrane and exert its antimicrobial action, either by puncturing a hole or by penetrating the target cell and inhibiting basic pathways. Our structural insight suggests that Slf may function as a classical bacteriocin by damaging the cell membrane or cell wall; however, it could also be a signalling molecule that initiates a programmed pathway to suicide or sporulation. According to the mathematical models of Slf-mediated toxicity, the Slf concentration is dramatically increased immediately adjacent to the colony front. Thus, although the production of Slf is induced by a sibling colony (via secreted subtilisin), the protein factor itself is derived from the host. Technically, this is closer to induced suicide than direct fratricide. The propensity of these Slf precursor homologues to enable specific control of bacterial growth is attractive to medicine and biotechnology and future research should seek to characterise the mechanism and specificity of action. Furthermore, the mechanism of proteolytic activation could provide an attractive mechanism for activating the peptide at the desired location.

### Accession numbers

Coordinates and structure factors have been deposited in the Protein Data Bank with accession numbers 5CIV, 5COM, 5CQV, 5COF and 5COG.

## Figures and Tables

**Fig. 1:**
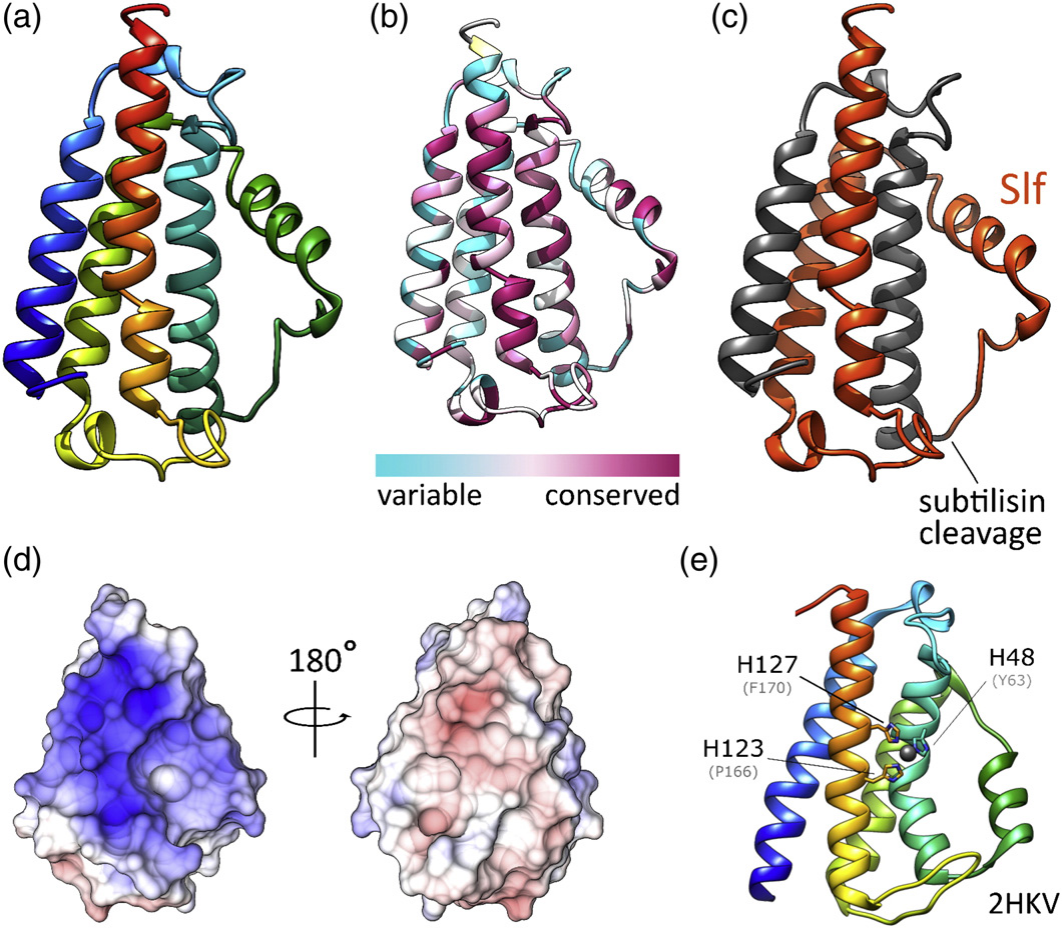
The X-ray crystal structure of DfsB from *P*. *dendritiformis*. (a) Cartoon rendering of PdDfsB [rainbow colours: N-terminus (blue) to C-terminus (red)]. (b) Conservation of residue identity within DfsB homologues. ConSurf was used to identify 250 sequence homologues (sequence identity: 35–95%) and determine the relative conservation for each position. The C-terminal helix is relatively well conserved, whereas the proposed subtilisin digest site is not. (c) Structure of PdDfsB indicating the location of processing by subtilisin. The resultant sibling lethal factor (Slf) polypeptide is coloured orange. (d) Surface representation of the electrostatic potential calculation of PdDfsB generated using the PDB2PQR server [13]. There is a strong positively charged patch on the front face of the protein, shown in same orientation as (a), whereas the rear face is mostly neutral or negatively charged. (e) Cartoon rendering of a member from the DinB family of proteins. The side chains for the histidine triad are shown as sticks and labelled with residue numbers. The bound nickel ion is shown as a grey sphere. Equivalent positions in PdDfsB are shown in parenthesis to highlight the lack of conservation for the metal binding site. The Pd*DfsB* gene was codon optimised for *E*. *coli* and synthesised by GeneArt (Life Technologies) with an N-terminal MRGSHHHHHHGS tag. The genes were ligated into pQE-30 using HindIII and BamHI restriction sites. The plasmid encoding PdDfsB was transformed into *E*. *coli* BL21 (DE3) cells. Cells were grown in Terrific broth medium at 37 °C until the cell density reached mid-log phase (OD_600_ = 0.6–0.8) when expression was induced by addition of IPTG to a final concentration of 0.5 mM. After incubation at 37 °C for 4 h, cells were harvested by centrifugation and frozen in liquid nitrogen. Cells containing PdDfsB were lysed in 50 mM sodium phosphate, 300 mM NaCl and 10 mM imidazole buffer using a cell disruptor (Constant Systems). The lysate was clarified by centrifugation at 18,000 rpm for 25 min. His-tagged protein was then captured by incubation with Ni-NTA resin (Qiagen) for 30 min at 4 °C. The resin was then gently centrifuged and transferred to a disposable 5-ml gravity column (Qiagen), washed with lysis buffer, followed by lysis buffer plus 25 mM imidazole. Purified target protein was eluted from the resin using lysis buffer plus 300 mM imidazole. PdDfsB was further purified and buffer exchanged into 10 mM Tris–HCl and 100 mM NaCl (pH 8.0) by gel filtration (Superdex 75 16/60, GE Healthcare). DfsB was crystallised in 0.1 M sodium formate, 0.1 M ammonium acetate, 0.1 M Sodium citrate tribasic dihydrate, 0.1 M sodium potassium tartrate tetrahydrate, 0.1 M sodium oxamate 0.1 M Tris, 0.1 M Bicine (pH 8.5), 12% PEG (*p*oly*e*thylene *g*lycol) 550 MME and 6% PEG 20,000. Crystals were harvested using nylon loops and flash-cooled in liquid nitrogen with no additional cryo-protection. SAD (single-wavelength anomalous dispersion) data collection was performed on the I03 beamline at Diamond Light Source, UK. SAD data were processed automatically by Xia2 using space group in the space group *P*2_1_2_1_2_1_ to a maximum resolution of 1.38 Å. The asymmetric unit contained a single copy of DfsB. Experimental phases were determined and initial model was calculated using AutoSol and AutoBuild, respectively (Phenix) [14], [15], [16]. This model was optimised manually using Coot [17] and refined using Phenix.Refine followed by Refmac with anisotropic *B*-factors. The final coordinates and structure factors were deposited in the Protein Data Bank as accession number 5CIV.

**Fig. 2:**
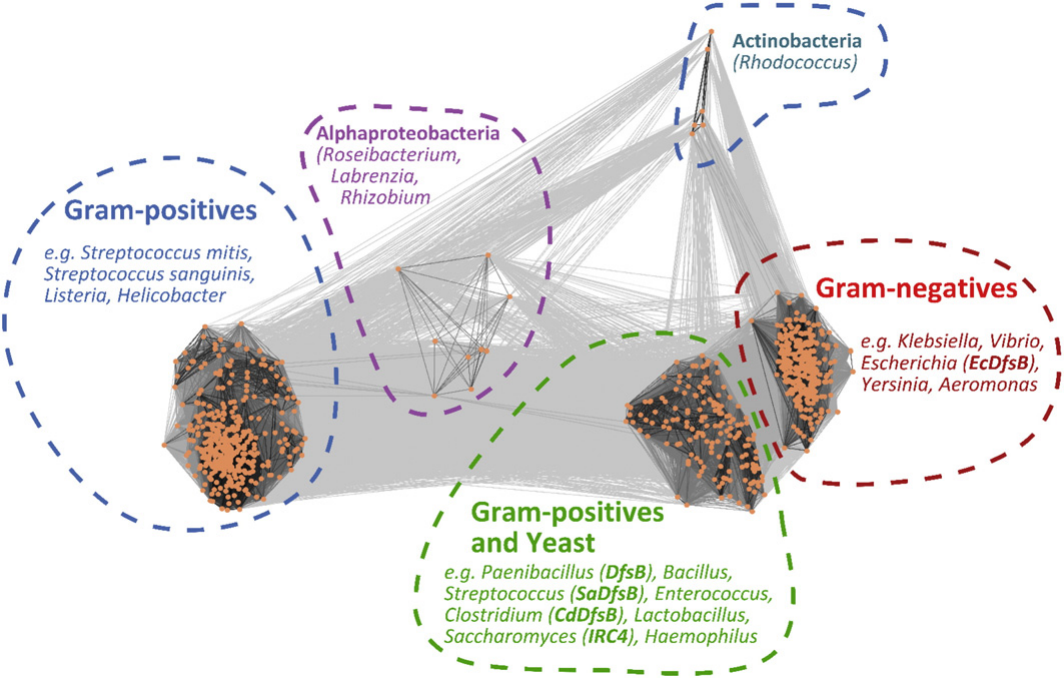
Phylogenetic relationships amongst DUF1706 family members. A subset of 819 sequences annotated as belonging to the DUF1706 family were subjected to pair-wise alignment and clustering by CLANS. Each sequence is represented by an orange dot, and each dot is connected by a line with shading proportional to individual pair-wise sequence identity. DUF1706 sequences clearly split into three main clades and two smaller clusters. Structures reported in this study are derived from the green and red clades. Protein sequences within the UniProt database annotated as belonging to the DUF1706 family (*n* = 1403) were filtered by removing identical sequences (*n* = 945) and retaining only sequences that had a length between 150 and 210 residues. The final 819 sequences were subjected to pair-wise sequence alignment by CLANS [18] using a *p* value of 1e^− 20^.

**Fig. 3:**
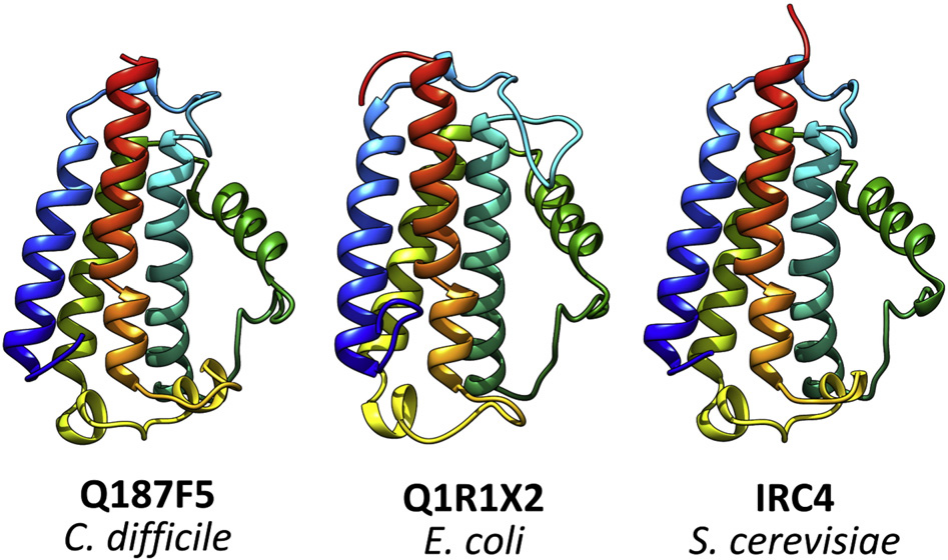
X-ray crystal structures of DfsB homologues from *Clostridium*, *Escherichia* and *Saccharomyces*. Each structure is shown in cartoon form, coloured in a rainbow gradient (N-terminus in blue to C-terminus in red). Structural alignment with DfsB revealed a close match in each case (CdDfsB = 0.81 Å, Ec = 1.2 Å and IRC4 = 0.74 Å). Cloning, protein production and purification for CdDfsB, ScIRC4 and EcDfsB were carried out using the same methods as for PdDfsB. Crystals of CdDfsB used for data collection were grown under the same condition as PdDfsB. Diffraction-quality crystals of EcDfsB were grown in 60 mM magnesium chloride hexahydrate, 60 mM calcium chloride dihydrate, 0.1 M imidazole, 0.1 M MES monohydrate (pH 6.5), 12.5% methyl-2,4-pentanediol, 12.5 % PEG 1000 and 12.5% PEG 3350. Diffraction-quality crystals for ScIRC4 were obtained in 0.09 M sodium nitrate, 0.09 M sodium phosphate dibasic, 0.09 M ammonium sulfate, 0.1 M sodium Hepes, 0.1 M Mops (pH 7.5), 12% PEG 550 MME and 6% PEG 20,000. Experimental phases were determined and models refined as for PdDfsB. Final coordinates and structure factors were deposited in the Protein Data Bank as accession numbers 5COM (CdDfsB), 5COF (EcDfsB) and 5COG (IRC4).

**Fig. 4:**
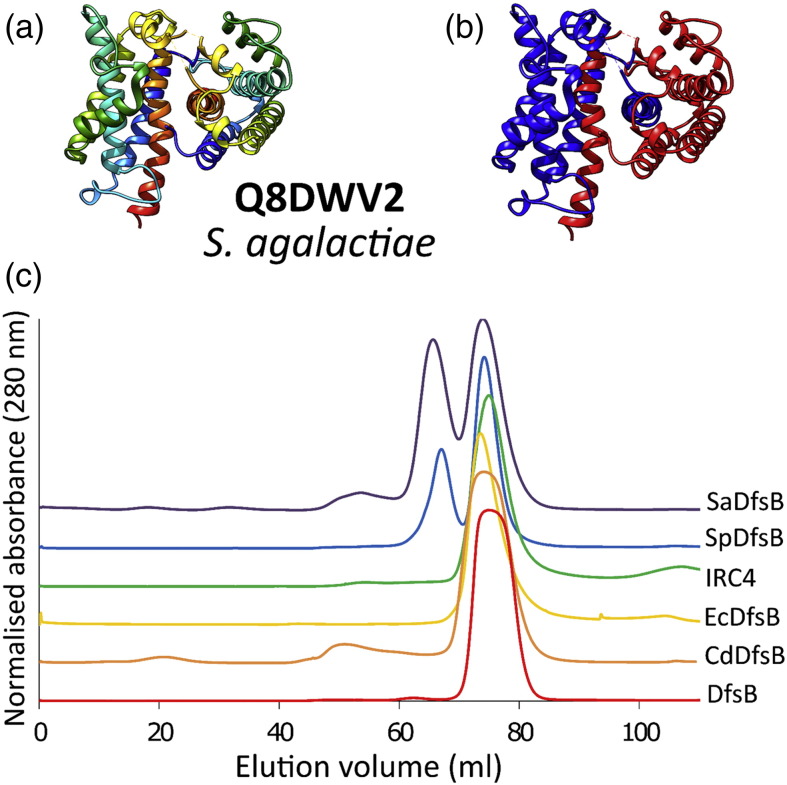
Structural diversity shown by a DfsB homologue from *S*. *agalactiae*. The X-ray crystal structure of dimeric SaDfsB is shown in cartoon form, coloured in a rainbow gradient (N-terminus in blue to C-terminus in red). (b) The C-terminal helix of each SaDfsB monomer flips out and inserts in the place of its partner within the dimer. The overall structure of SaDfsB remains very similar to DfsB (RMSD = 0.85 Å). (c) Gel-filtration profiles for DfsB and five homologues. The *Streptococcus* homologues (top panel) were unique in that they both displayed a mixture of monomers and dimers. DfsB and the other three homologues (lower panel) migrated as a monomer during gel filtration. Cloning, protein production and purification for the *Streptococcus* homologue SaDfsB was carried out using the same methods as for PdDfsB. SaDfsB migrated as a mixture of monomers and dimers and were effectively separated by the Superdex column. Only the dimeric fraction yielded diffraction-quality crystals in 0.12 M diethylene glycol, 0.12 M triethylene glycol, 0.12 M tetraethylene glycol, 0.12 M pentaethylene glycol, 0.1 M Tris, 0.1 M Bicine (pH 8.5), 12% PEG 550 MME and 6% PEG 20,000. The structure of SaDfsB was solved by molecular replacement using PHASER (within CCP4), with the structure of CdDfsB as a starting model. The final coordinates and structure factors were deposited in the Protein Data Bank as accession number 5CQV.

**Table 1:** Data collection and refinement statistics

	SeMet-DfsB	SeMet-CdDfsB	SeMet-EcDfsB	SaDfsB	IRC4
Wavelength (Å)	0.9788	0.9795	0.9795	0.91730	0.98
Resolution range (Å)	21.51–1.38 (1.43–1.38)	61.74–1.85 (1.87–1.85)	48.16–1.35 (1.42–1.35)	20.16–1.9 (1.97–1.9)	63.6–1.61 (1.67–1.61)
Space group	*P*212121	*C*121	*P*212121	*P*1211	*C*121
Unit cell dimensions (Å)	47.59, 47.68, 72.30	127.05, 50.30, 91.72	46.86, 48.16, 80.22	51.94, 65.93, 55.75	146.57, 38.69, 67.10
Angles (°)	90, 90, 90	90, 118.70, 90	90, 90, 90	90, 114.72, 90	90, 108.59, 90
Total reflections	200,349 (11,241)	566,321	541,538 (38,389)	90,560 (8920)	88,048 (8812)
Unique reflections	32,882 (2552)	80,903 (1985)	40,263 (5436)	26,515 (2622)	45,443 (4482)
Multiplicity	6.1 (4.4)	7 (5.5)	13.5 (7.1)	3.4 (3.4)	1.9 (2.0)
Completeness (%)	96.05 (75.39)	95.4 (69.4)	99.0 (93.3)	98.08 (97.73)	97.95 (98.10)
Mean *I*/σ(*I*)	17.38 (2.02)	15.8 (2.4)	15.1 (4.9)	17.09 (2.34)	10.01 (2.28)
Wilson *B*-factor	13.05	26.47	7.30	26.52	15.70
*R*-merge	0.07107 (0.7124)	0.073 (0.63)	0.149 (0.302)	0.04967 (0.4947)	0.04186 (0.2988)
*R*-meas	0.07752	0.105	0.160	0.05913	0.0592
CC1/2	0.999 (0.663)	0.979 (0.816)	0.992 (0.941)	0.999 (0.778)	0.998 (0.876)
*R*-work	0.1814 (0.2731)	0.1943 (0.27)	0.1151 (0.152)	0.1779 (0.2456)	0.1917 (0.2545)
*R*-free	0.1988 (0.2861)	0.1706 (0.31)	0.1385 (0.220)	0.2187 (0.2720)	0.2358 (0.2970)
Number of non-hydrogen atoms	1654	2958	1831	3048	3527
Number of macromolecules	1416	2947	1465	2866	2861
Number of ligands	—	15	12	16	13
Number of water	238	503	354	166	653
Protein residues	169	170	172	340	337
RMS (bonds)	0.006	0.011	0.011	0.008	0.006
RMS (angles)	1.02	1.08	1.48	1.10	0.97
Ramachandran favoured (%)	98	99.42	98.4	99	99
Ramachandran allowed (%)	2	0.58	1.6	1	1
Ramachandran outliers (%)	0	0	0	0	0
MolProbity Clashscore	2.15	3.74	4.06	5.04	5.10
Average *B*-factor	16.50	25.30	12.6	42.20	25.10
Macromolecules	14.70	23.20	9.71	42.10	22.00
Ligands	—	60.2	31.4	60.00	24.80
Solvent	27.50	36.9	26.0	42.70	39.10

Statistics for the highest-resolution shell are shown in parentheses.
