# Supplementary material for: Structures of the DfsB Protein Family Suggest a Cationic, Helical Sibling Lethal Factor Peptide
Source: J Mol Biol. 2016 Feb 13;428(3):554–60. doi: 10.1016/j.jmb.2016.01.013 (PMC4773401; doi:10.1016/j.jmb.2016.01.013)
Supplement: Supplementary file 1 — Supplementary material [file mmc1.pdf]

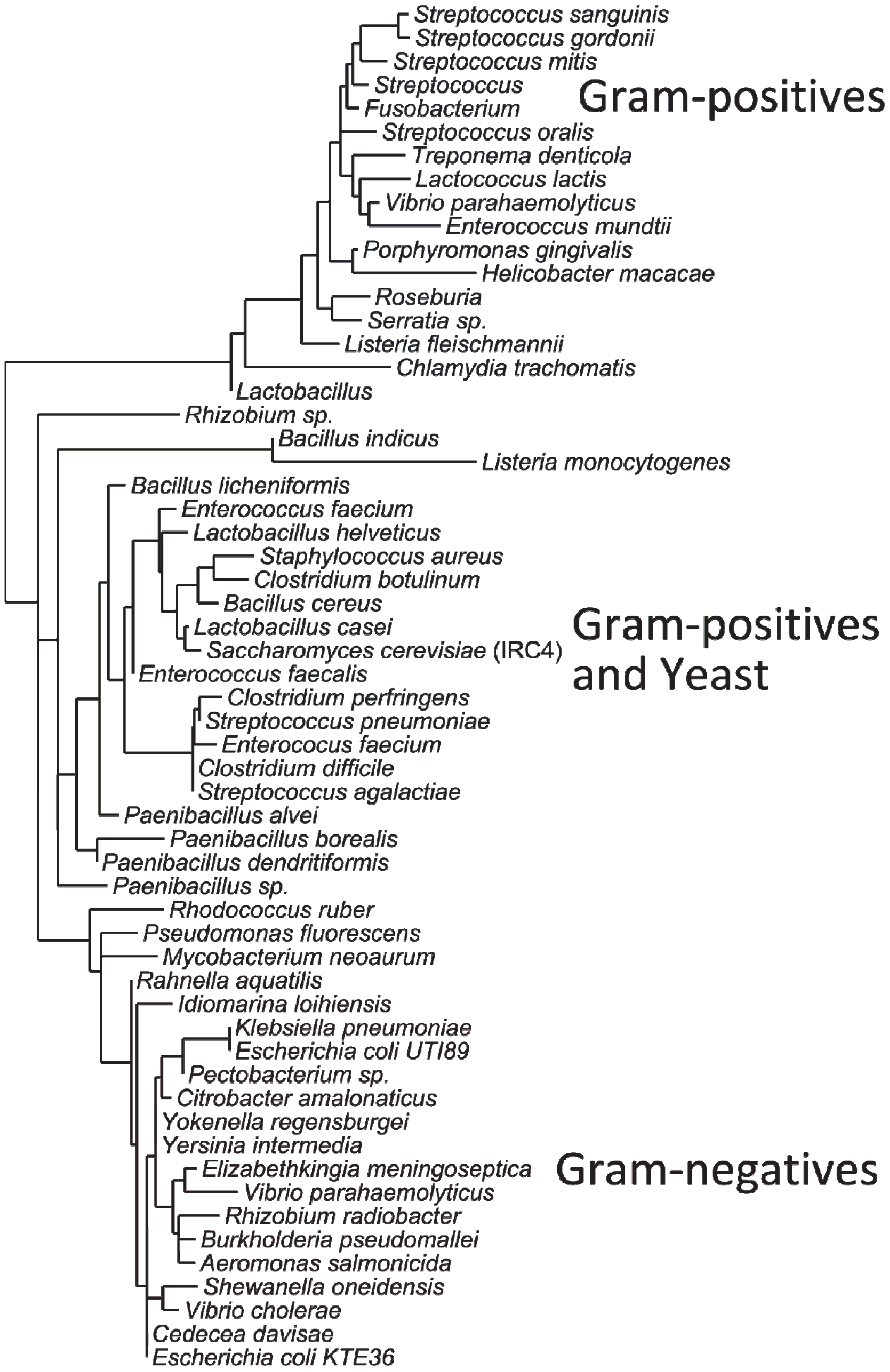

Supplementary Figure 1. Phylogenetic relationships between DUF1706 family members.
A set of 57 DUF1706 sequences with maximum 90% inter-sequence identity were aligned and used to create the phylogenetic tree. The highlighted groups correspond in colour to that of Figure 2.

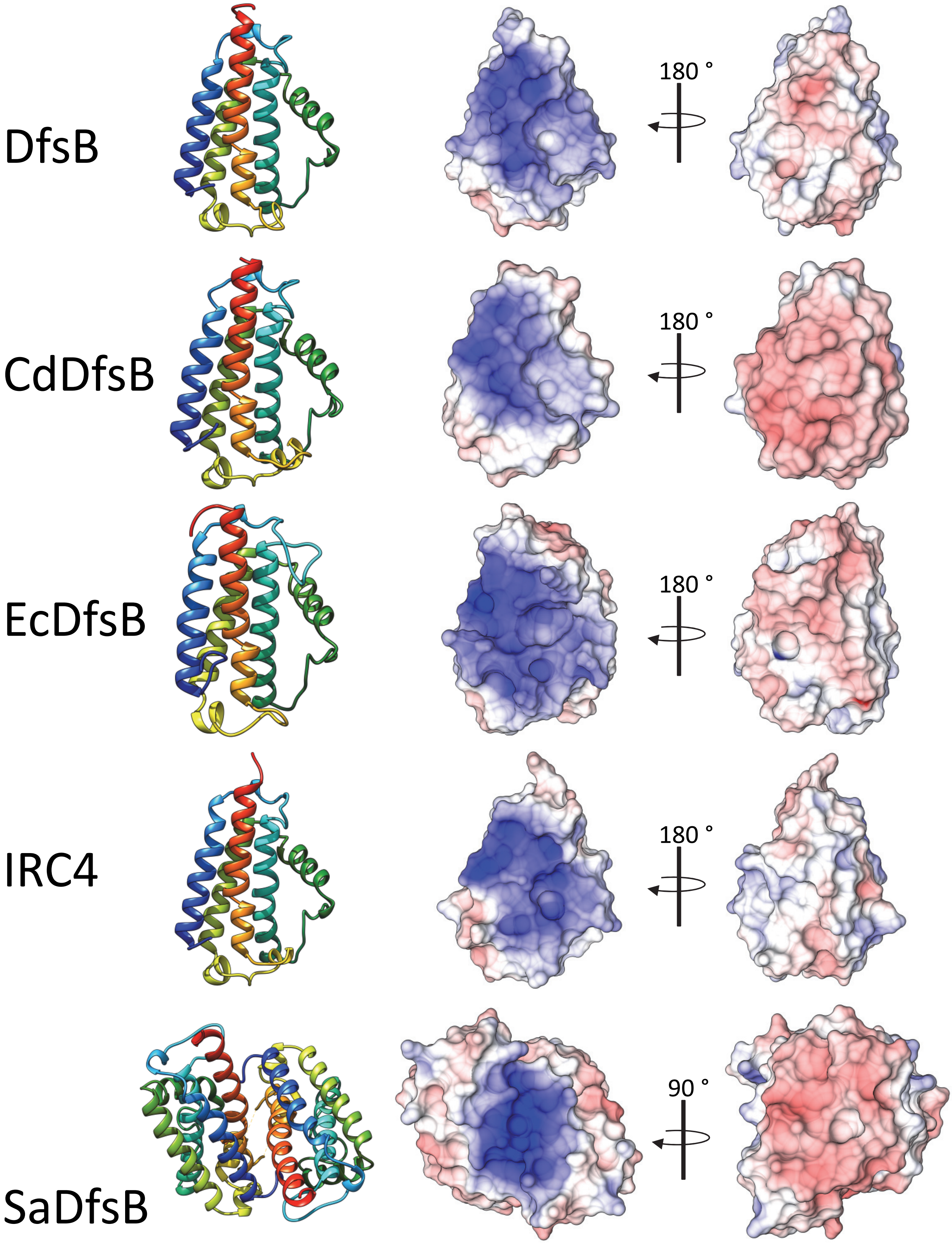

Supplementary Figure 2. Electrostatic surface potential of DfsB and homologues.
The APBS surface potential was calculated using the PDB2PQR server and visualised using Chimera. For reference, the cartoon structures in the left column are in the same orientation as the middle column with the conserved positive charge patch.
